# BRIC’s Growing Share of Global Health Spending and Their Diverging Pathways

**DOI:** 10.3389/fpubh.2015.00135

**Published:** 2015-05-06

**Authors:** Mihajlo B. Jakovljevic

**Affiliations:** ^1^Faculty of Medical Sciences, University of Kragujevac, Kragujevac, Serbia

**Keywords:** BRICS, health expenditures, long term, global health, trends, health care reform

## BRIC’s Growing Share in Global Wealth

Post-cold war developments and accelerated pace of globalization among many changes led to the creation of so called emerging markets. These classical national economies represent few among large number of developing world countries, which are distinguished by their exceptionally strong promise of rapid and long-term stable growth of gross domestic product. Either we assess it on nominal or purchase power parity (PPP) terms, four distinct economies obviously lay ahead all other rapidly developing global markets. Acronym BRIC (Brazil, Russia, India, China) forged to describe these countries brought glory to its creator Jim O’Neil, Goldman Sachs’ economist of the time (1). Since his first insight back in 2001 global recession (2) and ongoing developments were changing prospects for all four individual markets. Nevertheless, strong positive growth trend remained their common feature although with quite substantial differences in pace and balance of overall economy development (3). BRIC’s share in global wealth grew tremendously effectively quadrupling itself over past decade (4). Joint growth of this group of countries, heavily dominated by China, will remain long-term trend with clear forecasts at least up to the middle of twenty-first century (5).

## Consequences for National Health Systems of These Nations

Each one of BRICs countries enjoyed prolonged period of geopolitical stability. Local governments via different mechanisms succeeded to use welfare of the society to improve access and quality of health care (6). Rising middle classes contributed to the higher demand for pharmaceuticals and novel medical technologies, particularly in developed urban cores. Long-term neglect of rural populations, many of them living close to poverty line, finally led to more decisive polices to tackle these issues. Health insurance coverage recorded its first serious improvements in these regions (7). Affordability of medical care to ordinary citizens was spreading although not sufficiently to follow-up disproportionate rapid growth of out of pocket spending (8). This effectively meant some serious setbacks affecting health care access to the poor (9). Many of such issues remain high on local health policy agendas and unresolved so far. Another important obstacle in mammoth sized health sectors of these nations is delivery of cutting edge treatment options to the citizens. Local innovation rate remains quite modest compared to huge research and development investment particularly characteristic of People’s Republic of China (10). Promising signs are rapidly growing frequency of scientific publications in medicine, technology patents, and strengthening of local research capabilities in terms of human resources, institutional commitment, and capital investment into equipment. Although similar positive changes are clear in all four countries, China is once again surpassing all other BRIC members with its capacity and output (11).

## Total Health Expenditures among BRICs in Nominal and Purchase Power Parity Terms

**Figure 1:**
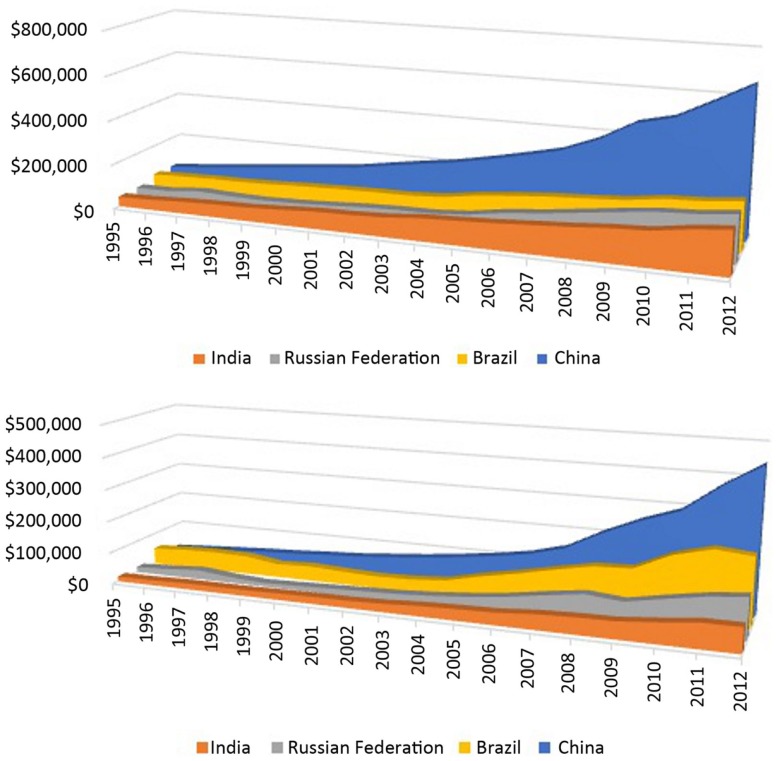
**Long-term trend on total national – level expenditure on health (THE) 1995–2012; Above: THE expressed in million current international $PPP (purchase power parity value); Beneath: THE expressed in million current US $ (nominal value); Source: Global Health Expenditure Database**.

Global health expenditure database (GHED) relying on national health accounts (NHA) system to track financial flows within national health systems of all World Health Organization (WHO) members across the globe was established since 1995 with latest official release of 2012 data (12). This is probably the most comprehensive single source allowing for international comparability of data. Observing these 18 years we might come to terms with many fine hidden patterns of health spending transformation that occurred worldwide and among the BRIC themselves. Global share of BRIC nations in total health expenditure (THE) grew from 4% ($108,938) to 12% ($858,193) in nominal terms ($USD) while change from 9% ($220,650) to 16% ($1,289,861) was even more profound in PPP terms. Joint health expenditure by BRIC nations succeeded to raise sixfold in less than two decades. Calculations of global health spending refer to 193 countries or political entities for whom complete records are available within GHED registry. Most surprising evidence comes from internal THE relationships among Brazil, India, Russian Federation, and China (Figure 1). Back in 1995, THE composition of BRICs in nominal terms was dominated by Brazil (31%) followed by China (29%) and approximately equal shares of Russia and India of 20%. Recent 2012 data point out to entirely different nominal THE landscape heavily dominated by China with 52%, followed by Brazil (17%), Russia (16%), and India (15%) all three very close to each other. THE expressed in $PPP reveals quite different picture. In 1995, Brazil held even 47% of joint spending while it was followed by China (24%), Russia (15%), and India (14%). If we observe percentage of gross domestic product (GDP) spent on health by individual countries it is easy to notice that only India remained at 4% level. Each of other three countries gained momentum of higher GDP proportion dedicated to health care today compared to situation 20 years ago. Such capital investment was led by Brazil (2.7% increase) followed by China (1.9) and Russia (0.9).

## Prospects of Retaining Long-Term Growth in Health Spending among the BRICs

All aforementioned data point out to the several important facts. In the beginning of observation period, Brazil was dominating the BRICs landscape both in terms of nominal and PPP and percentage of gross domestic product health spending (13). Over the course of years, Brazil remained on the lead only in terms of last one (14). It is THE expressed as percentage of GDP reached 9.31% topping the list with both scale of increased and its absolute value. India, regardless of huge increase in national welfare and economic output decided to forcibly maintain its expenditure at 4% of GDP (15). Respective amount available for various health programs became much larger anyway, so it recorded successes in expanding health insurance coverage and access to medical services (16). One important advantage of India compared to its three remaining counterparts is far younger population due to delayed population aging process in this large nation. Therefore, the burden of major prosperity diseases and elderly age remains significantly easier to cope with (17). Although India’s share in BRIC’s joint THE fell significantly in percentage terms we should not forget that scope of financial means disposable for health care actually quadrupled in same period in both nominal and PPP terms. Russian Federation recorded growth of THE in all terms over past two decades but its share of BRIC’s joint THE remained at the same level (18). Nevertheless, systemic health reforms and overall economic performance were developing in the last BRIC’s member faster than anywhere else (19). The BRIC’s composition of THE observed as national level spending from year to year becomes more and more dominated by China. This is still not the case with per capita spending where Russia’s THE per capita exceeds Chines three times ($1,474 PPP in 2012) and Brazilian ($1,109 PPP in 2012) more than twice. Many of microeconomic indicators and identified health system weaknesses point out that there is long ahead of Chinese health reforms (20). Regardless of some setbacks global multinational industry of pharmaceuticals and medicinal devices will target and support largest global markets (21). The potential of all BRIC nations, led by People’s Republic of China to absorb new medical technologies and further raise demand for medical goods and services will most likely remain high in the long run (22).

## BRIC’s vs. OECD’s Health Expenditures

Many forecasts actually point out to the growing competitiveness of BRICs compared to major OECD markets. OECD’s joint share of global health expenditure still far exceeds the one of BRICs although OECD/BRICs ratio of joint THE fell from 22 times in nominal terms in 1995 to 7 times in 2012. This same ratio expressed in PPP terms felt from ninefold larger THE in favor of OECD in 1995 to only fourfold larger THE in 2012. OECD’s proportion of global health spending fell from 91 to 81% in nominal terms and from 82 to 72% in PPP terms. The global trend of gains and losses in health spending clearly went in favor of largest emerging markets at the expense of mature, traditional high-income OECD economies (23). We should not forget that BRIC’s growth alone is not sufficient to explain existing differences. Significant part of these gains in national health budgets should be attributed to smaller N-11 emerging markets, South Africa and large number of middle- and low-income countries mostly situated in Asia, Eastern Europe, Latin America, and Africa (24). The global landscape of health care spending has clearly changed more in recent past than for the most of twentieth century (25).

## Beyond Tomorrow?

Health policy makers are aware they should stay precautious about newly built socioeconomic welfare of many developing countries. Their national capacities to direct investment and growing capacities into the most rewarding, evidence based and cost-effective medical procedures and drugs remain very limited. Knowledge-based resource allocation still has to make roots in health policy traditions of BRICs and other emerging nations (26). Health outcomes offer final judgment on success of health care delivery to the patients in needs. Longevity gains were indeed substantial while fall in neonatal, maternal mortality, and incidence rates of communicable diseases records continuous success in these countries (27). Nevertheless, life expectancy at birth and likelihood of healthy aging remain by far higher in high-income economies with Japan topping the list (28). Facing the upcoming burden of accelerated population aging will be particularly challenging in the emerging markets where such demographic transition was far more rapid compared to most of developed societies. Official UN forecasts tell us that China will be the fastest aging among large nations for many upcoming decades (29). Very similar changes, at slightly slower pace began happening much earlier in Russia followed by Brazil. Morbidity structure of BRICs, with partial exception of India, has already changed toward the one dominated by non-communicable prosperity diseases. All of BRICs share another important geographic determinant. They do have very uneven population distribution with exceptionally large rural areas remote to most specialty hospitals and university clinics. Development of rural network of medical facilities although traditionally stronger in Russia (30), presents particular challenge to China, India, and Brazil (31). Lack of willingness in local physicians and nurses to get employed in the country side far away from more attractive career prospects in large cities, presents another obstacle leading to effective shortages of professional staff (32). Common citizens inhabiting these areas usually earn less income than those living in rich industrial cities (33). Vulnerability to catastrophic household expenditure due to illness of family member is high (34). In line with these facts, out of pocket expenditure grew tremendously in all of BRICs from $67 PPP on average in 1995 to $276 PPP in 2012. Among several causes, widespread informal payments remain significant cost driver for ordinary people (35). Faced with so many ongoing challenges it would be very hard to present any reliable future forecasts for health care affordability and sustainable financing in BRICs (36). Whether their impressive long-term efforts will bring worthy fruits in population health will probably be fully visible in the second half of twenty-first century.

## Conflict of Interest Statement

The author declares that the research was conducted in the absence of any commercial or financial relationships that could be construed as a potential conflict of interest.
